# Psychometric properties of the Spousal Assault Risk Assessment from samples of people having perpetrated intimate partner violence

**DOI:** 10.1177/15248380241262275

**Published:** 2024-07-23

**Authors:** Victoria Allard, Tamsin Higgs, Maéva Slight

**Affiliations:** 1 University of Montreal, Canada; 2 International Center of Comparative Criminology, Montreal, Canada; 3 Institut national de psychiatrie légale Philippe-Pinel, Montreal, Canada

**Keywords:** domestic violence, assessment, predicting domestic violence

## Abstract

Since it was first published in 1995, the Spousal Assault Risk Assessment (SARA) Guide has become one of the most used and researched intimate partner violence (IPV) risk measures worldwide. Yet, no recent review has formally and systematically established the psychometric properties of this measure. Furthermore, the third version of the SARA (SARA-V3) was published in 2015, with no psychometric critique to date. This review aimed to provide an inclusive and exhaustive literature review of all psychometric properties (i.e., predictive validity, convergent validity, internal consistency, and inter-rater) of the SARA, including V3. A systematic search of 17 databases was conducted following Preferred Reporting Items for Systematic Reviews and Meta-Analysis (PRISMA) guidelines. Academic journals, book chapters, and gray literature were included but conference presentations were not. To be included studies had to report a psychometric property of any version of the SARA and be composed of individuals having committed IPV. The search identified 28 records published between 1997 and 2022. Results showed that although the literature on the SARA is mostly positive, it is much more varied in terms of both results and research quality than its widespread implementation might suggest. Most studies were conducted using case files in a research context with non-diverse samples, undermining ecological validity. Results for convergent and predictive validity were mostly positive. However, reliability statistics were under-researched and showed poorer results. Lastly, little research has gone into validating the SARA-V3, with what is available suggesting poorer reliability and validity than its predecessor. Practitioners are cautioned against transitioning to the newer version before further validation research has occurred.

Intimate partner violence (IPV) refers to violence occurring between current or former romantic or sexual partners, regardless of partner gender, living situation, or marital status (Beaupré, 2015). Roughly one in three women and one in four men will experience IPV at some point in their lifetime (National Center for Injury Prevention and Control, 2021; World Health Organization, 2023). IPV has thus been identified as a major global public health concern with tremendous impacts on the victims, their families, and society. These impacts were exacerbated during the COVID-19 pandemic, where IPV came to the forefront of public awareness (Evans, 2020). IPV is often recurrent, with 43% of victims reporting revictimization in a 12-month period and with recidivism rates averaging around 28% (Hanson et al., 2007; Rahman, 2018). With such high rates of recidivism, it is imperative that correctional services be able to accurately identify those most at risk of reoffending. Risk assessment measures are important to identify high-risk individuals and to adequately allocate resources. Additionally, by assessing an individual’s specific risk factors, they allow for tailored interventions, maximizing treatment effectiveness (Bonta & Andrews, 2017).

Paralleling the increase in IPV awareness, the last few decades saw an increase in the number of IPV-specific risk measures (Graham et al., 2021; Northcott, 2012; Svalin & Levander, 2020; van der Put et al., 2019). A recent review identified 28 different IPV-specific risk assessments (Maltais & Serin, 2023). Although numerous, these tools are not interchangeable as they were developed for different contexts, goals, and professionals (Messing & Thaller, 2013; Northcott, 2012). Furthermore, few of these have been extensively studied and shown to demonstrate strong empirical validity. One exception is the Spousal Assault Risk Assessment (SARA) (Kropp et al., 1995). Translated into over 10 languages and used in over 15 countries, the SARA is the most widely used and researched IPV-specific risk assessment measure (Helmus & Bourgon, 2011).

The SARA was developed for pretrial and presentencing evaluations as well as correctional intake and discharge but has also been used in a variety of contexts such as police screenings (Belfrage et al., 2012; Kropp et al., 1995). It contains 20 items scored on a three-point scale of *absent*, *partially or possibly present*, and *present*. The items cover four domains: Criminal history, psychosocial adjustment, spousal assault history, and index spousal assault offense. The manual recommends the use of both victim and perpetrator interviews, official reports, and clinical records to code the measure (Kropp et al., 1995). However, considering the many contexts where resources are limited, the manual still permits evaluators to use the SARA without all recommended information sources (Helmus & Bourgon, 2011).

The SARA was the first Structured Professional Judgment (SPJ) measure to have ever been developed (Kropp et al. 1994). Unlike actuarial risk assessment, which uses a mechanical approach to sum up risk factors, SPJ depends on professional discretion to evaluate risk according to a structured set of evidence-based guidelines (Borum 1996; Kropp, 2008). SPJ has been criticized for being less accurate in terms of predictive validity than their actuarial counterparts, yet recent reviews have found the two approaches to be roughly equivalent (Nicholls, 2016). The argument for SPJ is that it gives more flexibility to the evaluator than an actuarial tool while providing more validity, reliability, and transparency than an unstructured approach. When using the SARA, the evaluator first rates items according to guidelines and then considers all items to reach conclusions that describe the risk level as low, moderate, or high. These are referred to as summary risk ratings (SRR; Kropp & Hart, 1997). The SARA asks for two SRR: One for IPV and the other for general violence. The SARA also allows evaluators to deem items critical, meaning that the evaluator believes that their presence represents an imminent risk of harm for the specific case. Lastly, the SARA does not require extensive psychological training, allowing it to be used by a wide variety of professionals (Helmus & Bourgon, 2011). However, since it does rely heavily on professional judgment it does require the evaluator to have extensive knowledge of both IPV and risk assessment as well as having followed specialized training in the use of this tool (Kropp et al. 1995).

In 2015, Kropp and Hart published the third version of the SARA (SARA-V3), which includes notable changes from the previous version (Table 1). For one, critical items were removed as research showed they were rarely endorsed by evaluators and possessed poor inter-rater reliability (IRR) (Kropp & Hart, 2000). The SARA-V3 contains 24 items still coded on a three-point scale, now grouped into three sections: Nature of the IPV; perpetrator risk factors; and victim vulnerability factors. Rather than having different sections for past and index offenses, all items are now assessed for their presence both recently (i.e., in the past year) and in the past (i.e., prior to the past year). The perpetrator risk factors and victim vulnerability factors are also coded according to their relevance to case management (Kropp & Hart, 2015). SPJ is still used to establish SRR, however, this is done using a more descriptive approach with three ratings: Risk of severe harm, imminence, and case prioritization. Such an approach is argued to better communicate risk, as it is qualitative rather than probabilistic, and facilitates case management (Kropp, 2004). In other words, rather than giving a percentage likelihood or recidivism according to normed data, it talks about the nature of harm to future victims (i.e., severity), how soon it can be expected (i.e., imminence), and the amount of intervention required (i.e., case prioritization). The idea being that with this information, the evaluator can implement a more tailored case management plan than if solely a risk probability had been given.

**Table 1.** Comparison of original SARA and SARA-V3 items.

SARA Items	SARA-V3 Items
Spousal assault history	Nature of IPV factors
—	Intimidation
—	Threats
Past physical assault	Physical harm
Past sexual assault/sexual jealousy	Sexual harm
Past use of a weapon and/or credible threats of death	Severe IPV
—	Chronic IPV
Extreme minimization of denial of spousal assault history	—
Recent escalation in frequency or severity of assault	Escalating IPV
Past violation of “no contact” orders	IPV-related Supervision Violations
Alleged/most recent offense	—
Severe and/or sexual assault	—
Use of a weapon and/or credible threats of death	—
Violation of “no contact” orders	—
Psychosocial adjustment	Perpetrator risk factors
Recent relationship problems	Intimate relationships
Recent employment problems	Employment/finances
Victim of and/or witness to family violence as a child or adolescence	Trauma/victimization
Personality disorder with anger, impulsivity, or behavioral instability	Personality disorder
Recent substance abuse/dependence	Substance use
Recent psychotic or homicidal ideation/intent	Major mental disorder
—	Violent/suicidal ideation
—	Non-intimate relationships
—	Distorted thinking about IPV
Criminal history	General antisocial problems
Past assault of family members	—
Past assault of strangers or acquaintances	—
Past violations of conditional release or community supervision	—
—	Victim vulnerability factors
—	Barriers to security
—	Barriers to independence
—	Interpersonal resources
—	Community resources
—	Attitudes and behaviors
—	Mental health

*Note.* IPV = Intimate partner violence; SARA = Spousal Assault Risk Assessment.

In 2011, Helmus and Bourgon published a narrative review of 11 studies effectively summarizing the literature on the SARA. This review was geared toward clinicians and concluded that, although the research overall supported the use of the SARA, the study quality and results varied greatly from one study to the next. However, the review’s methodology was not reported and, as such, the extent to which studies were systematically identified, included or excluded, and quality assessed is unclear. More quantitative reviews (Hanson et al., 2007; Messing & Thaller, 2013) on IPV risk assessment have reported on the validity of the SARA. They report acceptable predictive accuracy (average weighted area under the curve (AUC) = 0.63; Messing & Thaller, 2013) and effect size (average weighted Cohen’s *d* = 0.47; Hanson et al., 2007). However, these were published over a decade ago, prior to the publication of the SARA-V3. As such, they do not establish whether the changes brought to the newer iteration result in better psychometric properties, or the extent to which the SARA-V3 has been validated. Meanwhile, more literature on the SARA has been published.

More recent meta-analytic reviews on IPV risk assessment have been published (i.e., Graham et al., 2021; Svalin & Levander, 2020; van der Put et al., 2019). Although informative these reviews included very few studies on the SARA due to their scope and restricted inclusion criteria and therefore do not paint a complete picture. One review only included studies where the measures were coded by professionals and therefore only two studies on the SARA met the criteria (Svalin & Levander, 2020). The most inclusive review (Van der Put et al., 2019) included only 10 studies on the SARA while a scoping exercise conducted at the outset of the present research indicated more studies are available. It should further be noted that these two meta-analyses only examined predictive validity and no other psychometric property. One study (Graham et al., 2021) did assess multiple psychometric properties, but again the inclusion criteria (i.e., English only, published prior to 2015, full-length versions only, etc.) limit the scope of the paper (*k* = 6). The present study aims to contribute to the literature by providing an up-to-date and exhaustive systematic review to synthesize the available data pertaining not only to predictive validity but all SARA (including V3) psychometric properties.

## Method

A systematic literature review was conducted following PRISMA guidelines (Page et al., 2021), using the 15 following bibliographic databases: Academic Search Complete, CINAHL, Criminal Justice Abstracts, CAIRN, ERIC, Érudit, IBSS, Medline, NCJRS, Pubmed, PsycINFO, Social Sciences Abstracts, Social Services Abstracts, Web of Science, Health and Psychology instruments and google scholar. A systematic search of the gray literature was also conducted using OpenGrey and Proquest Dissertation and Thesis Global. The search was conducted using the key words “Spousal Assault Risk Assessment” and a complementary search was also conducted in google scholar using more restrictive key words (“Spousal assault risk assessment” AND validity OR validation OR psychometric* OR reliability OR consistency OR accuracy OR test-retest OR inter-rater OR interrater”) to ensure no relevant literature was missed. After an initial screening of titles and abstracts, if full texts needed to be consulted that were not available online or though interlibrary loan, corresponding authors were contacted directly. An additional report that had not been identified through bibliographic searches, but which met the inclusion criteria, was sent by one of the contacted researchers. Further studies were included based on a backward literature search of the reference lists of selected studies and of Helmus and Bourgon’s (2011) review. Finally, a simple google search was conducted as an approximative verification of the search strategy.

### Inclusion/Exclusion Criteria

To be included, studies had to report at least one interpretable psychometric property of the SARA (i.e., a reliability or validity index). The sample had to be composed of individuals having committed IPV. There were no population-based exclusion criteria (i.e., gender, ethnicity, sexual orientation, age, country, etc.), to allow for the study to assess how the SARA performs within a range of diverse populations and to identify groups that are under-represented in the literature. Included studies consisted of peer-reviewed articles, book chapters, academic theses, and governmental reports. Secondary data, conference presentations, posters, and mediatic articles were excluded. All study designs were included (i.e., prospective, retrospective, or cross-sectional). All languages were included, and no date range was specified. Studies in languages other than English were translated using DeepL translation.

### Study Selection

**Figure 1.**
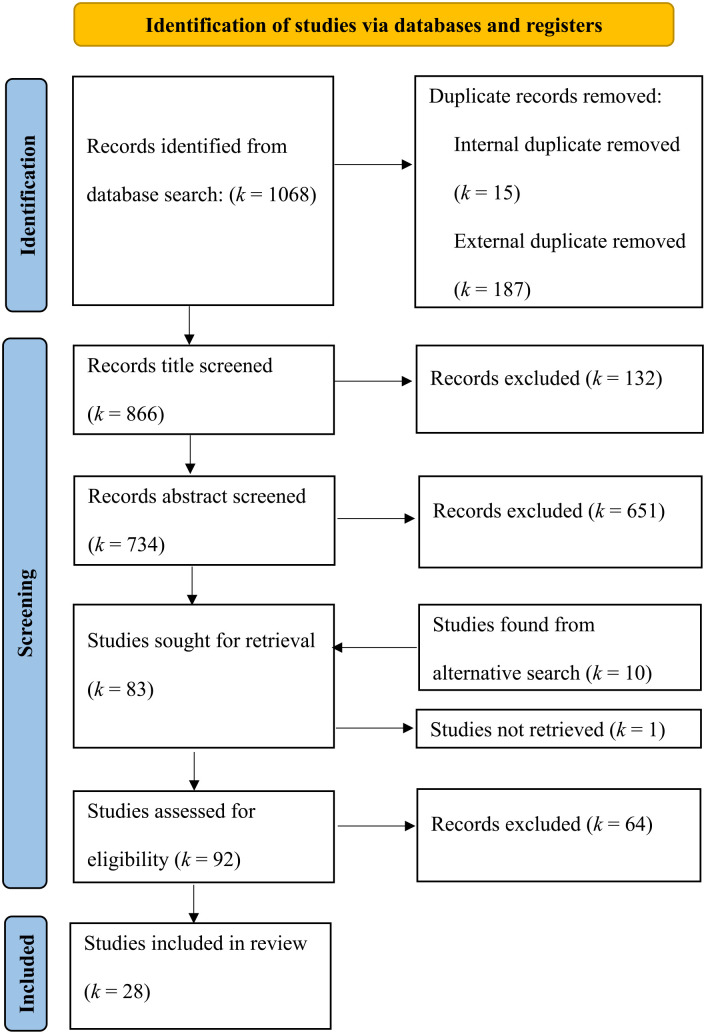
Flow diagram summarizing the systematic record screening process. *Note.* Flow diagram adapted from Page et al. (2021). *k* refers to the number of studies or records.

The database searches yielded 1,068 results, brought down to 866 after duplicate removal. Following a screening process for titles and abstracts and then full texts, a total of 28 studies were retained (Figure 1). To verify the screening procedure, IRR using Cohen’s kappa was calculated on approximately 10% of abstracts and full texts, resulting in kappa statistics of .73 and 1 respectively. An additional 14% of abstracts were dual-coded at different time points to assess inter-rater drift, bringing the overall kappa down to .64. This was greatly influenced by the small number of studies meeting inclusion criteria later in the screening process, giving high weight to disagreements. Overall percentage agreement remained high at 94%. All disagreements were resolved with a discussion between raters.

### Quality Assessment and Data Extraction

Studies were also coded using a quality assessment form to establish their empirical quality. The form was developed in line with Cochrane review guidelines (Ryan et al., 2013). The form contained questions evaluating the quality of the sampling procedure, measurements, methods, and outcome to establish the overall scientific rigor and lack of bias. Each study received an overall quality rating of either low, moderate, or high risk of research bias. IRR was determined based on 16 studies. Percentage agreement was used to assess reliability due to the small number of studies. Agreement on the items ranged from 38% to 94% with a mean agreement of 72%. Raters had 75% agreement for the final quality rating. All disagreements were resolved with a discussion between raters.

Using a data extraction form, each study was coded by the first author to extract publication details, aims, methods (i.e., participants, procedures, and measures), results, limitations, and future directions. Results were put in tables to compare studies and to synthesize data. Originally, predictive validity data was to be meta-analyzed. In the end, this was not achievable as the necessary statistics required to calculate effect sizes (Cohen’s *d*) were too infrequently reported. Corresponding authors were contacted but with a low reply rate. Instead, predictive validity was assessed using weighted summary AUC using MedCalc statistical software when sufficient information was reported.

## Results

The results represent 28 records across 22 studies published between 1997 and 2022. Some records were grouped together as they represent the same larger study which resulted in multiple articles with different results and subsamples. The list of all included studies and relevant descriptive information is summarized in Table 2. Most studies took place in Canada (*k* = 14), followed by the United States (*k* = 7), while the remaining studies are spread across Sweden (*k* = 2), Portugal (*k* = 2), Argentina (*k* = 1), and New Zealand (*k* = 1). Samples ranged from very small treatment studies (*n* = 45) to large correctional samples (*n* = 2,044). In almost all cases, the sample was composed entirely of men except for a few studies with mixed samples where the percentage of women ranged between 6% and 15% (Jung & Buro, 2017; Olver & Jung, 2017; Wong, 2008, 2010; Wong & Hisashima, 2008; Wong & Sadaya, 2011). In over half of the studies (54%) the assessment was done by researchers rather than field professionals. The context of assessment varied greatly from community police contact to intake for treatment programs to court assessments. Multiple studies (Ennis et al., 2017; Jung & Buro, 2017; Jung et al., 2022; Olver & Jung, 2017) used modified versions of the SARA. The Ennis et al. study (2017) simply coded items present or absent rather than using the three-point scoring. Olver and Jung (2017) used a 17-item version, while the two studies by Jung and Buro (2017) and Jung et al. (2022) used a 14-item version. Convergent validity with the full SARA was not established. These short versions are due to lack of information in case records. In fact, over half of the studies completed ratings solely using records (*k* = 14), rather than the recommended interviews and multiple information sources. Additionally, most studies scored the SARA in an actuarial manner, summing up the points obtained on the various items, rather than using the SRR. In fact, only eight studies specify the use of SRR.

**Table 2.** Descriptive Summaries of All Included Studies on the Psychometric Properties of the SARA Measures.

Study	SARA Version	Sample Size	Mean (SD)	Country	Sample Type	Study Design	Risk Evaluator	Information Source
Andres-Pueyo et al. (2008)	SARA	102	19.58 (6.88)	Spain	Court cases	Retrospective	Researchers	Case files
Arbach and Folino (2021)	SARA	125	14.11 (6.69)	Argentina	Court assessment	Prospective	Professionals	Interview and file review
Belfrage et al. (2012)	SARA	429	11.48 (6.08)	Sweden	Community police contact	Prospective	Police officers	Police records
Callan-Bartkiw (2012)	SARA	43	14.14 (7.87)	New Zealand	Community treatment program	Prospective	Researchers	Interview
Cunha and Gonçalves (2015)	SARA	45	—	Portugal	Treatment program	Prospective	—	Interview
Cunha and Gonçalves (2019)	SARA	172	—	Portugal	Inmates and community	Retrospective	Researchers	Interview and case files
Ennis et al. (2017)	SARA	105	—	Canada	Cases referred for threat assessment	Retrospective	Researchers	Police records
Glackman and Buchanan (2004a, 2004b)	SARA	2044	—	Canada	Corrections	Prospective	—	—
Grann and Wedin (2002)	SARA	88	20.47 (4.66)	Sweden	Forensic psychiatry	Retrospective	Researchers	Case files
Gray (2012)	SARA	94	18.15 (5.91)	Canada	Federal inmates referred for treatment	Prospective	Parole officers	Interview, informants, police reports, and criminal records
Heckert and Gondolf (2004)	SARA	499	—	United States	Treatment program (court-mandated)	Prospective	Researchers	Questionnaire and victim interview
Hilton et al. (2004)	SARA	589;100	3.11 (4.14)	Canada	Community police contact	Retrospective	Researchers	Police records
Hilton et al. (2008)	SARA	649	4.63 (4.94)	Canada	Community police contact	Retrospective	Researchers	Police records
Hilton et al. (2021)	SARA-V3	238	48.31 (14.28)	Canada	Charged cases referred for threat assessment	Cross-sectional	Researchers	Case files
	SARA	238	22.87 (5.77)	Canada	Charged cases referred for threat assessment	Cross-sectional	Researchers	Case files
Kropp and Hart (1997)	SARA	226	—	Canada	Community forensic treatment	Cross-sectional	Professionals	Interview
Kropp and Hart (2000)	SARA	2,681	—	Canada	Probationers and federal inmates	Retrospective	Correctional, mental health, and research staff	Interview and file review
Olver and Jung (2017) ^a^	SARA	300	12.00 (5.53)	Canada	Community police contact	Retrospective	Researchers	Police records
Jung and Buro (2017)	SARA	238	10.9 (5.00)	Canada	Community police contact	Retrospective	Researchers	Police records
Jung et al. (2022)	SARA	246	9.29 (4.77)	Canada	Community police contact (all with stalking history)	Retrospective	Researchers	Police records
Ryan (2016)	SARA-V3	97	24.88 (4.90)	Canada	Presentencing assessment	Retrospective	Researchers	Case files
	SARA	84	20.06 (6.21)	Canada	Presentencing assessment	Retrospective	Psychologists	Court assessment
Schafers et al. (2021)	SARA-V3	88	14.8 (5.24)	Canada	Treatment program (court-mandated)	Prospective	Researchers	Case files
Skilling and Nonemaker (2010)	SARA	498	—	United States	Misdemeanor offenders	Prospective	—	Court records
Williams and Houghton (2004)	SARA	434	9.40 (6.20)	United States	Arrested perpetrators	Prospective	Probation officers	Perpetrator and victim interview, criminal records
Wong and Hisashima (2008) ^b^	SARA	196	10.40 (3.21)	United States	Probationers	Prospective	—	—
Wong (2008)	SARA	196		United States	Probationers	Prospective	—	—
Wong (2010)	SARA	103	—	United States	Probationers	Prospective	—	—
Wong and Sadaya (2011)	SARA	198	10.03 (6.10)	United States	Probationers	Prospective	—	—

*Note.* SARA = Spousal Assault Risk Assessment.

As for the SARA-V3, although ample time has passed, only three studies investigated its psychometric properties (Hilton et al., 2021; Ryan, 2016; Schafers et al., 2021). Furthermore, none of these three studies used the SARA-V3 in its entirety. Hilton et al. (2021) did not include the relevancy items in their analyses, while Ryan (2016) and Schafers et al. (2021) scored the items for their presence overall, rather than past and recent behavior. Schafers et al. (2021) also used prior SARA assessments to inform item scoring on the SARA-V3, while Ryan (2016) excluded the victim vulnerability section as the information was too infrequently found in files.

### Reliability of the SARA

#### Internal Consistency

Internal consistency refers to the extent the different items in a measure are related to one other, for if the items are strongly related then they must all be reliably measuring the same latent construct. The most common measure of internal consistency is Cronbach’s alpha, where scores > .70 represent good internal consistency (Hinkin, 1998). In their validation study, the authors of the SARA (Kropp & Hart, 2000) reported good internal consistency (α = .78). However, this seems to be the higher end of the results with only Arbach and Folino’s (2021) finding a barely higher alpha (.79), while the rest range from α = .63 to .77, establishing the SARA’s internal consistency as moderately good. Results are more contradictory when it comes to the SARA-V3. Only two studies report its internal consistency: One as excellent (α = .87; Hilton et al., 2021) and the other as below threshold (α = .66; Ryan, 2016), making it hard to establish the true internal consistency of the newest version (See Table 3).

**Table 3.** Summary of Results for the Different Reliability Statistics of the SARA Measures.

Study	Actuarial vs. SRR	SARA Version	Internal Consistency	Inter-rater Reliability		Test-retest Reliability	Item Homogeneity	
			*a*	ICC	Kappa	ICC	MIC	CITC
Arbach and Folino (2021)	SRR	SARA	0.79	0.84	.30–1.00	—	—	0.09–0.58
Belfrage et al. (2012)								
	Actuarial	SARA	—	—	—	0.76	—	—
	SRR	SARA	—	—	—	0.45	—	—
Callan-Bartkiw (2012)								
	Actuarial	SARA	—	0.29–0.84	—	—	—	—
	SRR	SARA	—	0.18–0.28	—	—	—	—
Cunha and Gonçalves (2015)	Actuarial	SARA	0.77	—	.72–.96	—	—	—
Cunha and Gonçalves (2019)	Actuarial	SARA	—	—	.72–.96	—	—	—
Glackman and Buchanan (2004b)	SRR	SARA	—	—	—	—	—	0.28–0.45
Grann and Wedin (2002)	Actuarial	SARA	—	0.85	.30–1.00	—	—	—
Hilton et al. (2021)								
	Actuarial	SARA	0.66	0.70	−.03–.87	—	—	—
	Actuarial	SARA—V3	0.87	0.83	.01–1.00	—	—	—
Kropp and Hart (1997)								
	Actuarial	SARA	—	0.92	—	—	0.15	—
	SRR	SARA	—	0.80	—	—	—	—
Kropp and Hart (2000)			0.78	—	—	—	—	—
	Actuarial	SARA	—	0.84	—	—	—	—
	SRR	SARA	—	0.57–0.63	—	—	—	—
Jung and Buro (2017) ^a^	Actuarial	SARA	—	—	.12–.80	—	—	
Olver and Jung (2017) ^a^	Actuarial	SARA	0.74	0.84	—	—	—	—
Jung et al. (2022)	Actuarial	SARA	—	—	.12–.80	—	—	—
Ryan (2016)			0.66	—	—	—	0.10	0.07–0.59
	Actuarial	SARA-V3	—	0.85	—	—	—	—
	SRR	SARA-V3	—	0.40–0.68	—	—	—	—
Schafers et al. (2021)	Actuarial	SARA-3	—	—	—	0.75	—	—
Williams and Houghton (2004)	Actuarial	SARA	0.71	—	—	—	—	—
Wong and Hisashima (2008) ^b^	Actuarial	SARA	0.65	—	—	—	—	—
Wong (2010)	Actuarial	SARA	—	—	—	—	—	0.37–0.47^c^
Wong and Sadaya (2011)	Actuarial	SARA	0.63	—	—	—	—	—

*Note.* Inter-rater reliability for actuarial total scores and summary risk ratings is reported using inter-class correlations (ICC). Item level inter-rater reliability is reported using the kappa statistic of the lowest and highest-scoring items. CITC = corrected item-total correlation; MIC = mean inter-item correlation; SARA = Spousal Assault Risk Assessment; SRR = summary risk ratings.

a The article by Jung et al. (2022) and Jung and Buro (2017) both use subsamples of the Olver and Jung (2017) sample. Their results should be interpreted together.

b The study by Wong and Sadaya (2011) represents a longer follow-up of a subsample from Wong and Hisashima (2008) study. Results should be interpreted together.

c Only reports the five highest-scoring items.

#### Item Homogeneity

Related to internal constancy is item homogeneity, which establishes the unidimensionality of the items within the measure (Tavakol & Dennick, 2011). This property is rarely reported in risk assessment literature as risk is traditionally defined as a multidimensional construct and coefficients are thus typically lower than on other clinical measures. Item homogeneity can be measured using both mean inter-item correlation (MIC) and corrected item-total correlations (CITC). The literature suggests that 0.15 is the lowest possible acceptable value (Clark & Watson, 2019), while other authors will establish the limit at 0.20 (Piedmont, 2014). Additionally, items should not exceed 0.50 as this would imply redundancy among the items. Kropp and Hart (1997) report the MIC of the SARA as 0.15. Ryan (2016) establishes the MIC of the SARA-V3 at 0.10 for the whole measure, 0.10 for perpetrator risk factors, and 0.21 nature of IPV section (See Table 3). These results indicate poor to barely adequate item homogeneity.

Arbach and Folino (2021) report CITC as ranging from 0.15 to 0.58, except for one item (*victim and/or witness of family violence as a child or adolescent*) which had a CITC of 0.09. Glackman and﻿ Buchanan (2004b) on the other hand reports a smaller range of scores (0.28–0.45). It should be noted that CITC start to be considered acceptable as of 0.20, although greater scores are preferable (Everitt, 2002). Wong (2010) also reports the five items with the highest CITC: *victim and/or witness of family violence as a child or adolescent* (0.47), *past physical assault* (0.47), *past assault of family members* (0.46), *recent relationship problems* (0.37), and *recent escalation in frequency or severity of assault* (0.37). As for the SARA-V3, the CITC ranged from 0.07 (*presence of sexual harm*) to 0.59 (*relevance of non-intimate relationships*) with most items falling between 0.21 and 0.54 (Ryan, 2016; See Table 3).

#### Inter-Rater Reliability

When it comes to risk assessment, IRR assures the measure’s resistance to bias in coding. IRR is essential to avoid either over or under-rating the evaluee, especially in high-stakes forensic contexts (Higgs et al., 2018). The strength of the agreement for final ratings is typically calculated using inter-class correlations (ICC), with values above 0.60 regarded as good and above 0.75 as excellent (Fleiss, 1986). In 1997, Kropp and Hart first report excellent IRR agreement for both actuarial scoring (ICC = 0.92) and SRR (ICC = 0.80). A later study reports similar results for actuarial scoring (ICC = 0.84) but much lower scores for SRR: ICC = 0.57 for low vs. moderate vs. high classifications, ICC = .63 for low-moderate vs. high classification (Kropp & Hart, 2000). In general, actuarially scoring of the SARA tends to result in higher IRR (ICC = 0.70–0.85) than using an SPJ approach (ICC = 0.40–0.84). These results exclude the results of Callan-Bartkiw’s (2012) study which found very low rater agreement as assessed under multiple contexts. When the second rater was coding the data only using the first rater’s notes this resulted in an ICC of 0.29 for actuarial scoring and 0.18 for SRR. As such the low agreement is more likely a reflection of incomplete information than of a true issue of reliability. Interestingly though is that the highest scores were not obtained when notes and recordings were combined (ICC = 0.36 for actuarial; ICC = 0.10 for SRR), but rather when the second rater only had access to the recording (ICC = 0.84 for actuarial; ICC = 0.28 for SRR). As for the SARA-V3 two studies looked at its inter-rater agreement, finding excellent scores (ICC = 0.83 and 0.85; Hilton et al., 2021; Ryan, 2016). Only one study looked at the different SRR: ICC = 0.40 for case prioritization, ICC = 0.41 for imminent violence, ICC = 0.68 for serious harm (See Table 3).

IRR can also be calculated at the item level and is typically done using Cohen’s kappa, where .60 and above is interpreted as good agreement while values greater than .80 are excellent (McHugh, 2012). There was a lot of variability in kappas not only between studies but also across the items. For example, Hilton et al. (2021) report a kappa of −.03 for the SARA’s worst item (*Personality disorder with anger, impulsivity, or behavioral instability*) and one of .87 for the best item (*Recent substance abuse/dependence*), while the mean kappa was .43. In the same study the SARA-V3’s items ranged from 0.01 (*Past escalating IPV*) to 1.00 for multiple items (*past and recent chronic IPV*, *non-intimate relationships past and recent*), with a mean kappa of .54 and .51 for past and recent items respectively. Other studies on the SARA had a similarly wide range (Arbach & Folino, 2021; Grann & Wedin, 2002; Jung & Buro, 2017; Jung et al., 2022) except for two studies (Cunha & Gonçalves, 2015, 2019) where kappas showed much less variability (*k* = .72–.96; see Table 3).

#### Test-Retest Reliability

Test-retest reliability looks at the consistency of a measure across time. Like IRR it is also calculated using ICCs. Test-retest reliability may not be the most adequate measure of reliability when it comes to risk assessment considering that these measures contain risk factors that are dynamic in nature and, therefore meant to fluctuate over time. As such a lack of agreement between assessments may reflect changing risk rather than a lack of reliability. One study did look at the agreement on SARA-V3’s perpetrator risk factors pre- and post-treatment, ICC = 0.75 (Schafers et al., 2021). Although this result can be interpreted as a form of test-retest it is hard to know whether agreement would have been higher had there not been any intervention. Another study looked at the SARA’s reliability between first and second police contact, with excellent scores for the measure when scored in an actuarial fashion (ICC = 0.76), but with moderately low scores for the SRR (ICC = 0.45).

### Validity of the SARA

#### Convergent Validity

One way to establish the validity of a measure is to see how strongly it correlates with other measures of the same, or related, construct. Multiple studies have looked at how the SARA and SARA-V3 correlated with other IPV-specific risk measures (See Table 4). Most notably the Ontario Domestic Assault Risk Assessment (ODARA; *k* = 12). For the SARA, correlations ranged from weak (*r* = .35) to strong (*r* = .74), with all but two studies falling above 0.60 (Arbach & Folino, 2021; Ennis et al., 2017; Gray, 2012; Hilton et al., 2004, 2008, 2021; Jung & Buro, 2017; Olver & Jung, 2017). One study compared the correlation with the ODARA for both the actuarial total score (*r* = .72) and SRR (*r* = .64; Arbach & Folino, 2021). The SARA-V3 and the ODARA were moderately correlated (*r* = .45–.59; Hilton et al., 2021; Ryan, 2016; Schafers et al., 2021). Multiple studies (*k* = 5) also looked at the correlation between the SARA and the Domestic Violence Screening Instrument scores, finding a strong correlation (*r* = .54–.74; Callan-Bartkiw, 2012; Skilling & Nonemaker, 2010; Williams & Houghton, 2004; Wong & Hisashima, 2008), except for one study that found a weak correlation (*r* = .16; Wong & Sadaya, 2011). Furthermore, actuarial and SRR were found to be comparable, *r* = .54 and .57 respectively (Williams & Houghton, 2004). The SARA and the SARA-V3 were also compared to its short-version, the Brief-Spousal Assault Form for the Evaluation of Risk (B-SAFER) (*r* = .59 and .70 respectively; Hilton et al., 2021). It is unsurprising that the B-SAFER was more highly correlated with the SARA-V3, as they were both designed with the same three-section format, with items coded in a similar fashion, and at times assessing the same risk factors. As for the Domestic Violence Risk Appraisal Guide, it was found to be weakly correlated with the SARA (*r* = .28; Grann & Wedin, 2002) but had a good correlation with the SARA-V3 (*r* = .57; Ryan, 2016). Ryan (2016) also established the correlation between the SARA-V3 and the Danger Assessment (*r* = .45). The SARA was strongly correlated with the Family Violence Investigative Report (*r* = .75; Jung & Buro, 2017; Olver & Jung, 2017). Although family violence typically is not limited solely to IPV, in this sample it was only used when the violence was done to an intimate partner.

**Table 4.** Summary of the Convergent Validity of SARA Measures.

Study	SARA Version	ODARA	DVSI	B-SAFER	DVRAG	FVIR	VRAG	PCL	GSIR	Other
Arbach and Folino (2021)										
Actuarial score	SARA	.72***	—	—	—	—	—	—	—	—
Summary risk rating	SARA	.64**	—	—	—	—	—	—	—	—
Callan-Bartkiw (2012)										
Actuarial score	SARA	—	.74*	—	—	—	—	—	—	—
Summary risk rating	SARA	—	.52*	—	—	—	—	—	—	—
Ennis et al. (2017)	SARA	.38***	—	—	—	—	—	—	—	—
Grann and Wedin (2002)	SARA	—	—	—	—	—	—	.59**	—	.46** (HCR-20:H)
Gray (2012)	SARA	.35**	—	—	.28**	—	—	—	—	—
Hilton et al. (2004)	SARA	.60**	—	—	—	—	—	—	—	—
Hilton et al. (2008)	SARA	.57***	—	—	—	—	—	—	—	—
Hilton et al. (2021)										
	SARA	.60**	—	.59**	—	—	—	—	—	—
	SARA-V3	.48**	—	.70**	—	—	—	—	—	—
Kropp and Hart (2000)										
Actuarial score	SARA	—	—	—	—	—	.29	.43***	-.07	—
Summary risk rating	SARA	—	—	—	—	—	.11	.34**	.01	—
Jung and Buro (2017) ^a^	SARA	.72***	—	—	—	.75***	—	—	—	—
Olver and Jung (2017)	SARA	.74***	—	—	—	.75***	—	—	—	—
Ryan (2016)	SARA-V3	.45***	—	—	.57***	—	—	—	—	.45***(DA)
Schafers et al. (2021) ^b^	SARA-V3	.56***/.59***	—	—	—	—	—	—	—	—
Skilling and Nonemaker (2010)	SARA	—	.67***	—	—	—	—	—	—	—
Williams and Houghton (2004)										
Actuarial score	SARA	—	.54***	—	—	—	—	—	—	—
Summary risk rating	SARA	—	.57***	—	—	—	—	—	—	—
Wong and Hisashima (2008) ^c^	SARA	—	.54**	—	—	—	—	—	—	.43**(LSI-R)
Wong and Sadaya (2011)	SARA	—	.16*	—	—	—	—	—	—	—

*Note.* Results reported as correlation coefficients. B-SAFER = Brief-Spousal Assault Form for the Evaluation of Risk; DA = Danger Assessment; DVRAG = Domestic Violence Risk Assessment Guide; DVSI = Domestic Violence Risk Instrument; FVIR = Family Violence Information Report; GSIR = General Statistical Information Recidivism Scale; HCR-20 = H-HCR-20 Historical subscale; LSI-R = Level of Service Inventory-Revised; ODARA = Ontario Domestic Assault Risk Assessment; PCL = Psychopathy Checklist-Revised or Screening Version; SARA = Spousal Assault Risk Assessment; VRAG = Violence Risk Assessment Guide.

a Scores = pre-treatment/post-treatment.

b The article by Jung et al. (2022) and Jung and Buro (2017) both use subsamples of the Olver and Jung (2017) sample. Their results should be interpreted together.

c The study by Wong and Sadaya (2011) represents a longer follow-up of a subsample from the Wong and Hisashima (2008) study. Results should be interpreted together.

* *p* < .05. **p < .01. ***p < .001.

Fewer studies looked at the relationship between the SARA measures and general and violent risk assessments. One found weak and non-significant associations with both the Violence Risk Appraisal Guide and the General Statistical Information Recidivism Scale (Kropp & Hart, 2000). Moderate correlations were found with the Hare Psychopathy Checklist (*r* = .34–.59), as well as the historical section of the HCR-20 (*r* = .46) and the Level of Service Inventory – Revised (*r* = .43; Grann & Wedin, 2002; Kropp & Hart, 2000; Wong & Hisashima, 2008).

#### Predictive Validity

Predictive validity is considered throughout the literature on risk assessment to be the “gold standard” validity index. It establishes the degree to which a scale does what it set out to do: Predict recidivism. Many statistical methods are used to establish predictive validity but the most popular is the receiver operator characteristic (ROC) AUC. In short, the ROC represents the rate of true-positive, individuals identified as high risk that do recidivate, in contrast to false-positives, those identified as high risk that do not recidivate, and this at all classification thresholds (Wilber, 2022). Once this curve is plotted, the AUC represents the likelihood that a randomly selected subject that recidivated scored higher than a randomly selected one that did not (Messing & Thaller 2013). In other words, AUCs represent the proportion of correct classifications with a score of 1 representing perfect classification, while an AUC of 0.50 is equal to chance (Wilber, 2022). A scale is thus considered to have modest predictive ability from 0.60 to 0.70, moderate predictive validity as of 0.70, while anything above 0.80 is considered excellent (Finch et al., 2017). Other analyses to establish predictive validity include but are not limited to, correlations, regressions, and *t*-tests comparing groups of recidivists and non-recidivists.

##### ROC Analyses

The predictive validity of the SARA using the AUC has been widely researched (*k* = 16). Most studies have looked at IPV-specific predictive validity using actuarial scoring (*k* = 14). Wong and Sadaya’s (2011) results were rather poor, predicting recidivism at less than chance. In other studies results ranged from chance (AUC = 0.50) to good predictive validity (AUC = 0.74), demonstrating a large amount of variability in the results (Arbach & Folino, 2021; Olver & Jung, 2017). When taken together this resulted in an weighted summary AUC of 0.63. The SARA seems to have overall better results where violent and general recidivism were concerned, although this has been assessed by fewer studies (*k* = 4 and 8 respectively). For most studies assessing violent recidivism, predictive validity ranged from 0.66 to 0.74, while for general recidivism these values ranged from 0.63 to 0.78 (Jung & Buro, 2017; Olver & Jung, 2017; Schafers et al., 2021; Williams & Houghton, 2004; Wong & Hisashima, 2008; Wong & Sadaya, 2011). The one exception was Gray (2012), which found values just barely above chance (AUC = 0.53 and 0.52). Altogether this resulted in an weighted summary AUC of 0.67 and 0.72 for violent and general recidivism, respectively. One study also established the SARA’s ability to predict general recidivism in a subsample of women (*n* = 45, AUC = 0.86) and found that the addition of women to the overall sample did not lower predictive accuracy of any recidivism outcome (Olver & Jung, 2017). Lastly, although Skilling and Nonemaker (2010) found that the predictive validity of the SARA preformed above chance for both IPV-specific and general recidivism, they did not specify their results (see table 5).

**Table 5.** Summary of the Predictive Validity of SARA Measures Using Area Under the Curve (AUC).

Study	SARA Version	Sample Size	Recidivism Source	Recidivism Definition	Mean Follow-up (months)	Total Score (AUC)			Summary Risk Ratings (AUC)		
						IPV recidi.	Violent recidi.	General recidi.	IPV recidi.	Violent recidi.	General recidi.
Arbach and Folino (2021)	SARA	122	Criminal records	New case	8	0.50	—	—	0.64	—	—
Belfrage et al. (2012)	SARA	429	Police records	Subsequent police contact	18	0.63	—	—	0.57	—	—
Callan-Bartkiw (2012)	SARA	36	Police records	Domestic disputes	9	0.72	—	—	0.78	—	—
Grann and Wedin (2002) ^a^	SARA	56–88	Police records	Convictions	45	0.52–0.65	—	—	—	—	—
Gray (2012)	SARA	94	Correctional and police records	Charges or convictions	65	0.60	0.54	0.52	—	—	—
Heckert and Gondolf (2004)	SARA	499	Partner report	Re-assault or abuse	15	0.64	—	—	—	—	—
Hilton et al. (2004)	SARA	100/589	Police records	Subsequent violent assault	5	0.54/0.64	—	—	—	—	—
Hilton et al. (2008)	SARA	649	Police, corrections, and criminal record	Any incident of assaultive behavior	61	0.59	—	—	—	—	—
Kropp and Hart (2000)	SARA	102	—	Charges or convictions	—	—	—	—	0.70	—	—
Jung and Buro (2017) ^b^	SARA	198	Criminal records	Charges/convictions	40	0.68/0.74	0.66/0.72	0.75/0.76	—	—	—
Olver and Jung (2017)	SARA	300	Criminal records	Convictions	40	0.74	0.74	0.78	—	—	—
Schafers et al. (2021)	SARA-V3	88	Court database	Charges	15	—	0.68–0.70	0.75–0.77	—	—	—
Skilling and Nonemaker (2010)	SARA	468	Court database	Convictions	96	>0.50	—	>0.50	—	—	—
Williams and Houghton (2004) ^c^	SARA	1,465	Criminal records	Arrests	18	0.65	—	0.70	0.65	—	0.71
Wong and Hisashima (2008) ^d^	SARA	249	—	Arrests	3	0.61	—	0.63	—	—	—
Wong and Sadaya (2011)	SARA	198	—	Arrests	36	<0.50	—	0.58	—	—	—
Weighted summary AUC^e^						0.63	0.67	0.72	0.65	—	—

*Note.* AUC = area under the curve; IPV = Intimate partner violence; SARA = Spousal Assault Risk Assessment.

a AUC assessed at 6-, 12-, 24-, and 60-month follow-ups with sample sizes of 88, 87, 83, and 56 participants respectively.

b The article by Jung et al. (2022) and Jung and Buro (2017) both use subsamples of the Olver and Jung (2017) sample. Their results should be interpreted together.

c William and Houghton’s (2004) SRR are a weighted SARA scores based on the imminence summary risk rating.

d The study by Wong and Sadaya (2011) represents a longer follow-up of a subsample from the Wong and Hisashima (2008) study. Results should be interpreted together.

e AUCs from the following articles were not included in the weighted summary AUC analysis as they were not reported in a suffieciently detailed manner: Heckert and Gondolf, 2004; Hilton et al., 2008; Skilling and Nonemaker, 2010; Williams and Houghton, 2004; Wong and Hisashima, 2008; Wong and Sadaya, 2011. The article by Schafers et al. (2021) was also excluded since it uses the SARA-V3 and therefore represents a different measure. Additionally, studies where multiple AUCs were reported (Grann and Wedin, 2002; Hilton et al., 2004; Jung and Buro, 2017) had each result entered into the analysis seperately.

Five studies looked at the IPV-specific predictive validity of the SARA using SRR, with results ranging from 0.57 to 0.78 and an weighted summary AUC of 0.65 (Arbach & Folino, 2021; Belfrage et al., 2012; Callan-Bartkiw, 2012; Kropp & Hart, 2000; Williams & Houghton, 2004). This indicates that actuarial scoring methods and SRR are equivalent in terms of predictive accuracy. Lastly, only one study looked at predictive ability of SRR for general recidivism, finding moderate predictive accuracy (AUC = 0.71; Williams & Houghton, 2004).

Surprisingly, considering the importance of predictive validity and the near decade since the SARA-V3 was published, only one study has looked at its predictive validity (Schafers et al., 2021). This one study is limited by the fact that it only assessed violent (AUC = 0.68–0.70) and general recidivism (AUC = 0.75–0.77), but not IPV recidivism, and its small sample size (*n* = 88).

##### Other Predictive Validity Analyses

Some studies looked at the predictive validity of the SARA using methods other than the AUC. One study found that a modified short form of the SARA was moderately correlated with subsequent IPV convictions (*r* = .23, *p* < .001) and charges (*r* = .23, *p* < .001), while similar results were found for violent recidivism (*r* = .28 and .26, *p* < .001), and strong correlations were found for general recidivism (*r* = .46 and .43, *p* < .001; Jung & Buro, 2017). A subsequent study in the same sample (Olver & Jung, 2017) used Cox regression survival analyses and saw that the Criminal History and Psychosocial Adjustment subsections of the SARA both significantly predicted IPV recidivism, while the Psychosocial Adjustment section was the only one to demonstrate incremental validity over the ODARA. When recidivists and non-recidivists were compared using a t-test, no significant difference was found for the SARA total score or part one (psychosocial and general violence factors), but a significant difference was found for the Spousal Assault History section, the number of risk factors, and the number of critical items (Kropp & Hart, 2000). Wong (2008) examined the records of 196 Hawaiian offenders identified as at risk of committing IPV over a 3-year period. They found that there was a statistically significant difference in both the IPV and general recidivism rates between low-medium risk offenders (defined as a score of eight or lower on the SARA) and high-risk offenders (scores greater than nine). Another study sought to validate the Spanish version of the SARA using a 12-month retrospective study of 102 couples where the victim officially filed IPV charges (Andres-Pueyo et al., 2008). They found that the SARA correctly identified 85% of recidivists and those individuals scoring above the mean (*M* = 19.58) were almost six times more likely to recidivate than those scoring below the mean (OR = 5.77, 95% CI = 2.4–13.8). It should be noted that although these last two studies (Andres-Pueyo et al., 2008; Wong, 2008) both sought to establish a cut-off score for the SARA, they did so in two very different samples (low-risk Hawaiian sample vs. high-risk Spanish sample), arriving at very different cut-off scores. Therefore, if the SARA is to be used in such an actuarial manner, which is against its intended use, it is imperative that clinicians refer to appropriate reference groups.

##### Item Level Predictive Validity

While most studies focused on how the entire scale performs, some authors also analyzed how the individual items inform risk assessment. Glackman & Buchanan (2004b) found that the SARA’s items had overall weak (*r* = .07; *Past assault of family members*) to moderate (*r* = .24; *Past violation of conditional release and/or community supervision*) correlations with reoffence. Using principal components analysis, they also found that the SARA’s items heavily loaded onto six components, suggesting redundancy among the items, especially considering that the six-component model correctly classified recidivists at a similar proportion to the total scale: 74.1% versus 73.8%. Meanwhile, Wong and Hisashima (2008) found that only seven of the SARA’s 20 items were significantly related to recidivism with almost all these items being statistic risk factors related to past criminality. The one exception was *the presence of a personality disorder with anger, impulsivity, or behavioral instability*. One further study looked at the relationship between item endorsement and recidivism using odds ratios and found that the partial or full presence of seven items negatively predicted recidivism (Grann & Wedin, 2002). The items with the strongest relation to recidivism were *extreme minimization of denial of spousal assault history* (OR = 8.18, 95% CI = 0.12–65.58), *personality disorder with anger, impulsivity, or behavioral instability* (OR = 7.57, 95% CI = 1.64–34.96), and *past physical assault history* (OR = 4.06, 95% CI = 0.49–33.94).

## Discussion

Due to the prevalence of IPV and the widespread use of the SARA in its assessment, it is essential that both researchers and clinicians be informed of the literature on the SARA, its validity and reliability (See Table 6), as well as its limitations and implications (See Table 7). This review summarizes the literature on the SARA and SARA-V3 to paint the most complete picture of the tools’ psychometric properties. We identified 28 records reporting on the SARA measures with variable, although generally favorable, results. The literature was considerably lacking when it came to reporting reliability statistics. Surprisingly few studies reported Cronbach’s alpha, even though this is commonly considered standard practice when presenting a measure, while measures of item homogeneity were almost entirely omitted from the literature. Furthermore, no study reported other measures of internal consistency such as MacDonald’s omega, which is generally superior to Cronbach’s alpha. Although these statistics tend to be lower for risk assessments than other, more unidimensional, measures they are still relevant in establishing the functioning of a scale and should be included in future studies. As for the IRR of the SARA, when it came to actuarially determined total scores, reliability was found to be excellent but was a lot poorer when it came to SRR. This result may be because fewer studies used SRR, however, it can also be the result of the more subjective nature of this final risk score. Although limited to the results of only two studies, test-retest reliability was also found to be superior when the measure was scored in an actuarial manner as opposed to when SRR were used. It is possible that SRR better captures the dynamic nature of risk, and thus fluctuates more over time, however here too the difference may be the result of the rater subjectivity and bias involved in SPJ. Further studies are needed to establish the true cause of this discrepancy.

**Table 6.** Summary of Critical Findings.

• Twenty-eight studies investigated the psychometric properties of the SARA measures, most of them taking place in North America. • Slightly over half the studies (54%) had the SARA coded in a research context, while only seven were coded by professionals in a clinical context. • Only three studies looked at the psychometric properties of the SARA-V3 and found them to be inferior to the SARA-V2. • Studies did not consistently report reliability coefficients. Those that did report found variable results. Overall, internal consistency was found to be moderate, mean inter-item correlation to be poor, and corrected item totals ranged from very poor to excellent depending on the item. • Overall, the SARA measures demonstrated good IRR when scored in an actuarial manner but much lower reliability when SRR were used to communicate risk. IRR at the item level showed a great amount of variability, with multiple items being less than adequate. • The SARA measures showed strong convergent validity with multiple IPV measures, most notably the ODARA, but also the DVSI, the DVRAG, DA, and the B-SAFER. The SARA measures also showed good convergent validity with general and violence measures such as the PCL, HCR-20, and LSI-R. • The SARA was found to have acceptable if somewhat modest, predictive accuracy when predicting IPV recidivism (weighted summary AUC = 0.63 for actuarially summed total score, AUC = 0.65 for SRR). Predictive accuracy was slightly higher for violent (AUC = 0.67) and general (AUC = 0.72) recidivism. • Only one study investigated the predictive accuracy of the SARA-V3. The SARA-V3 predicted violence (AUC = 0.68–0.70) and general recidivism (AUC = 0.75–0.77) with moderate accuracy. • Few studies analyzed validity at the item level. Those who did found that most of the SARA’s items do not directly inform IPV recidivism risk assessment.

*Note.* AUC = area under the curve; B-SAFER = Brief-Spousal Assault Form for the Evaluation of Risk; DA = Danger Assessment; DVRAG = Domestic Violence Risk Assessment Guide; DVSI = Domestic Violence Risk Instrument; HCR-20 = Historical Clinical Risk Management-20 – Historical subscale; IRR = Inter-rater reliability; LSI-R = Level of Service Inventory-Revised; ODARA = Ontario Domestic Assault Risk Assessment; PCL = Psychopathy Checklist-Revised or Screening Version; SARA = Spousal Assault Risk Assessment; SRR = summary risk ratings.

**Table 7.** Implications for Practice, Policy, and Research.

Implications for research
• More research should be conducted on the reliability of the SARA. Reliability statistics such as Cronbach’s alpha, Macdonald’s Omega, mean inter-item correlation, and inter-rater reliability need to be assessed and reported. • Efforts should be made to standardize how predictive validity is analyzed and reported in research. Although most studies report the AUC, some use different less robust approaches making comparison difficult. • Studies should also systematically report recidivism rates, as well as the mean SARA score of the sample, recidivists, and non-recidivists. This would allow for meta-analysis to be conducted allowing for a more comprehensive understanding of the literature. • More studies should test the individual contributions of the measure’s items, not just in terms of predictive validity and IRR, but also utilizing item-specific analyses such as Item Response Theory. • More research needs to be conducted on the SARA-V3 to establish its validity as only three studies have investigated this since its publication. • Researchers should also focus on verifying the field validity of the SARA measure as most studies use the SARA in a research context, coded from records by researchers with sections or items omitted. Few studies have validated the psychometric properties of the SARA measures according to their intended use. • Future research should evaluate predictive validity at multiple time points to establish the ability of the SARA measures to assess imminent, short, and long-term risk. • More effort should be made to validate the SARA measures in diverse groups such as ethnic minorities, native populations, women, and the LGBTQ+ community.
Implications for practice and policy
• Although the SARA is overall empirically supported, its psychometric properties are somewhat more modest than its widespread use would imply. Its limitations should be kept in mind during assessment. • The SARA had acceptable but modest predictive accuracy. Clinicians should bear in mind that there remains a certain margin of error when assessing clients. • The SARA is an SPJ measure meant to assist with case management. The use of SRR was found to be equivalent to when items are summed up actuarially in terms of predictive validity. SRRs can therefore be used to clinically inform case management without compromising predictive accuracy. • Although the SARA had acceptable predictive accuracy, many of its items did not. Only a handful of items showed a strong association with recidivism. This should be kept in mind when prioritizing treatment goals. • Clinicians should be wary of switching over to the SARA-V3 until more validation research has been conducted. • Clinicians should keep in mind that the SARA measures are not currently validated in minority groups (i.e., ethnic minorities, native populations, women, LGBTQ+). These groups can present risk factors in a different manner. Clinicians utilizing these measures in these groups should be aware of their realities and take this into account when using these measures, while still maintaining a SPJ approach.

*Note.* AUC = area under the curve; IRR = inter-rater reliability; SARA = Spousal Assault Risk Assessment; SPJ = Structured Professional Judgment; SRR = summary risk ratings.

The SARA was found to have high convergent validity with other IPV-specific risk assessments. This was especially the case with the ODARA, a highly validated actuarial IPV risk assessment. The correlations were weaker when it came to general and violence risk assessment. This can be seen as further proof of the validity of the SARA, as it correlates more strongly with measures of the same constructs than with measures of a similar construct. However, meta-analytic studies have pointed out that IPV-specific measures do not outperform general measures (Hanson et al., 2007; van der Put et al., 2019). As such, even if the items differ in nature these measures should arrive at similar scores.

The SARA was found to have acceptable accuracy in predicting recidivism, although some studies showed less favorable results and the weighted summary AUC was somewhat modest. Additionally, certain limitations need to be kept in mind. For one, the results from the different studies were compared directly. These results are not all equivalent as studies differ in terms of methodology and how recidivism was defined. Almost all the studies assessed recidivism using official records, which underestimates true recidivism rates. Records also varied in where they were obtained (i.e., police, court, or criminal records) representing different stages of the criminal justice process. Similarly, the definition of recidivism also varied greatly, from any indication of assaultive behavior to a new conviction. It goes without saying that the further down the legal process the definition is, the more likely it is to underestimate recidivism since victims may decide not to report the incidents, charges may be dropped, or plea deals may entail a non-guilty verdict for certain charges. Only one study used victim reports, representing the most likely recidivism rates, and found only modest predictive accuracy (AUC = 0.64; Heckert & Gondolf, 2004). Furthermore, there was a lot of variability in the length of follow-up, ranging from 3 to 65 months. This may be less of a limitation with IPV as recidivism tends to occur rather quickly compared to other forms of violence, like sexual violence, where recidivism often occurs many years down the line (Harris & Hanson, 2004; Quann, 2006). Nevertheless, studies with short follow-up periods, less than 6 months, may not accurately portray the true validity of the measure. The same goes for studies with small sample sizes. The minimum sample size for a validation study is typically regarded as 100 participants (Boateng et al., 2018). Lastly, although this study tried to include other ways to establish predictive validity aside from the AUC, these are much harder to interpret considering the variability in both statistical methods and their relevance. It is doubtful that a simple comparison of recidivism rates establishes validity to the same extent that the AUC does. Therefore, some standardization in how predictive validity is defined, measured, and reported would be beneficial (Graham et al., 2021).

Although the SARA seems to perform adequately as a whole, the few studies that analyzed the measure at the item level found that these were overall rather poorly related to IPV recidivism. Another review analyzing items across multiple measures also found that only three of the SARA’s items seem to significantly predict IPV recidivism (Maltais & Serin, 2023). The items that tend to relate to recidivism are static in nature, as is consistent with the literature (Caudy et al., 2013). The fact that the individual items of the SARA are not predictive, while the total score and SRR are, raises several concerns as to why that is. Perhaps the individual risk factors are not predictive but an overall poorer functioning, as reflected by a collection of risk factors regardless of their nature, does increase the chance of IPV recidivism. An argument can be made for the continued use of the SARA’s final rating to inform risk prediction as this is overall validated by the literature. However, one should be wary when focusing on the individual items. This also raises an issue regarding case management, which is the aim of the SARA. Since its dynamic risk factors are not clearly risk related, it is debatable whether they constitute valid intervention targets. Additionally, the item capturing personality disorders was the dynamic item most related to recidivism but was also one of the items with the lowest IRR, highlighting a different issue at the item level. No study has used analyses specifically meant for item level analyses such as item response theory (IRT; Bertrand & Blais, 2004). New to the field of criminology, IRT is well established with psychoeducational measures and studies have established its relevance in the context of criminal behavior (Giguère & Lussier, 2016; Giguère et al., 2023; Jose et al., 2012; Osgood et al., 2002). Yet IRT has never been used to develop or validate any IPV risk assessment measure.

The poor validity of the SARA’s items may also be due to the age of the measure. Helmus and Bourgon stated in 2011 that the SARA’s items were based on outdated literature and that this may in turn translate into poorer accuracy. The present review echoes this sentiment. The changes to the SARA-V3 were intended to reflect the progress in both the IPV and SPJ literature and therefore should translate into better psychometric properties (Ryan, 2016). However, the literature so far does not permit to establish if this is the case. Only three studies looked at the SARA-V3. This lack of literature on the SARA-V3 is both unexpected and concerning. It is true that research takes time, but in the near decade since its publication, more literature could have been expected. This may represent a reluctance form both researchers and professionals to start using the new version. This is possibly due to limitations in the design of the SARA-V3 which from experience, is complex and takes a long time to code and administer. Although it only contains 24 items, the fact that they are coded on multiple criteria (past, recent, and relevance) makes coding more laborious. Furthermore, rating items based on time frames may not always be practical or even feasible in certain contexts (recent items if the individual was incarcerated, the case took a long time to get to court, or if timeline information is limited). Additionally, the victim vulnerability section is difficult to assess when only the perpetrator or case records are available, which is the most common research scenario. The fact that this measure is resource intensive, in terms of both time and information required, may explain why so little research has been dedicated to it. Until more validity research is conducted, it would be premature for clinicians to migrate to the SARA-V3.

### Limitations in the Literature

The present review further identified multiple areas that are lacking in the quality of literature concerning the SARA’s psychometric properties. Most studies cited small sample size as a limitation. Many studies coded the SARA solely from records, and thus had to omit items due to missing information or used modified versions of the measure. Poor quality studies may translate into inflated or deflated results, resulting in a wide range of scores on almost every property assessed. Only a minority of the studies used the SARAs how they were intended. That is the full measure, without omissions, coded using both interviews and files by professionals using SPJ and SRR. These studies should thus be given more weight by readers as they represent the accuracy of the SARA in real-world conditions. However, in research, this is rarely how the SARA is administered. Only a handful of studies (*k* = 7) had the SARA administered by a field professional (i.e., psychologist, probation officer, police officer, social worker). This raises concerns for the field validity of the SARA and SARA-V3. Studies were also much more likely to use multiple information sources when the SARA was administered by a professional in a more realistic context. It should be noted that the greatest levels of predictive validity (AUC > 0.70) came from studies collecting data in a research context. The opposite can be seen with IRR. However, most of the IRR between professionals come from studies published by the SARA’s authors (Kropp & Hart, 1997, 2000). It may be speculated that these professionals received higher quality training on the measure than evaluators in other studies. Furthermore, a number of studies had very short follow-up periods and almost all studies only assessed predictive validity at one time point. Future studies should follow the example of Grann and Wedin (2002) and assess recidivism at multiple time points (e.g., 6, 12, 24, and 60 months) as this would allow for the predictive accuracy of the SARA measure to be established on the imminent, short, and long-term. This would be particularly relevant to the SARA-V3 as one of the SRR is specific to imminent risk.

Additionally, most of the studies took place in North America and all of them in the Western world. There is currently no data on how the SARA measures might perform in eastern populations, who generally have a different cultural understanding of intimate relationships, gender roles, and IPV (Ozaki & Otis, 2017). Even within the numerous studies from North America none looked at how the SARA preformed within different ethnic groups. In Canada specifically, indigenous populations are recognized to have their own distinct criminogenic pathways and risk factors. It is often argued that measures valid for the general population may not be valid for indigenous populations (Shepherd, 2016; Shepherd & Lewis-Fernandez, 2016). Nevertheless, no study has investigated this with the SARA. Another understudied population is women. Although a few studies did include women in their samples, they were often in small numbers. Only Olver and Jung (2017) looked at any psychometric properties while controlling gender. Yet, they were only able to report the predictive validity for general recidivism. During the review process, one study was identified that failed to meet the inclusion criteria but did administer the SARA in a women sample with a 20-month follow-up (Storey et al., 2012). In this study, women were evaluated using either the SARA (*n* = 52) or the B-SAFER (*n* = 54), but recidivism rates were too low for the AUC to be calculated (*n* = 5). The authors do report that three recidivists were classified as low risk and only one as high risk, suggesting a tendency toward false negatives. A larger sample is required to confirm this statistically. Lastly, no study looked at LGBTQ+ relationships even though research has shown that same-sex partners experience IPV at similar rates to opposite-sex partners (Gehring & Vaske, 2017).

### Present Study Strengths and Limitations

The primary strength of this study is its wide scope and large inclusion criteria, as it managed to include more studies than any prior review. By reviewing all psychometric properties, including at the item level, this review paints the most complete picture of the literature on the SARA currently available. Nevertheless, this review could have been even more inclusive. For one, it did not include conference proceedings. This was due to difficulties obtaining unpublished conference papers. For example, Helmus and Bourgon (2011) included three conference papers that we were unable to locate. However, since conference proceedings often lead to published papers, or otherwise include incomplete information, we believe this limitation to have only minimally impacted the results.

A second strength of this study is that it did not exclude studies in languages other than English. Although the study selection process only yielded a small number of non-English language papers (*k* = 2), the present review provides a more global understanding of the literature than if only English language studies had been included. On the other hand, a translation software was used. Some studies on the use of DeepL for academic papers have been published, but its use with various languages still requires further research (Takakusagi et al., 2021; Volkart et al., 2018; Zulfiqar et al., 2018). It is possible that errors occurred due to translation. Furthermore, although this study used a systematic approach, with inter-rater procedures and standardized data extraction forms to minimize human error, one cannot fully rule out the possibility of such an error occurring. The present methodology also does not counteract the file drawer problem, where studies with poor results tend to go unpublished (Rosenthal, 1979). This is partially mitigated by the inclusion of gray literature; however, readers should still regard the present results as an overestimation of the true properties of the SARA.

Lastly, this review did not include any meta-analyses of psychometric properties reported. This issue was partially counteracted by the inclusion of weighted summary AUC statistics. Nevertheless, the lack of detail and consistency in reporting psychometric properties is concerning for the literature. This makes it harder for studies to be compared to one another and for data to be interpreted comprehensively. Future validation studies should publish more details of the analysis. This includes the various reliability statistics but also recidivism rates and mean scores for all participants as well as recidivists and non-recidivists separately. This key and simple information was lacking in almost all studies, making a meta-analysis of predictive validity impossible as effect sizes could not be calculated.

## Conclusion

The present study substantially builds upon Helmus and Bourgon (2011) in establishing the psychometric properties of the SARA measures. The additional literature that has been reviewed concludes that the SARA performs acceptably, if modestly, in predicting IPV. Yet, there are notable limitations in the body of literature. Although the validity of the SARA has been extensively researched, research looking at its reliability is sparser. There are also several limitations in the quality and generalizability of the research literature. One solution for this is to produce higher quality and standardized research, including researching validity at the item level.

The failings of the SARA were meant to be corrected with the SARA-V3. Yet, in the near decade since its publication the SARA-V3 has barely been researched. When it was many items were frequently omitted. This seems to indicate either reluctance or difficulty in utilizing the SARA-V3 in research. Although the SARA-V3 is a promising innovation, clinicians should be mindful of its lack of validation before using it, especially since results so far seem to indicate poorer psychometric properties than those of its predecessor.

Nevertheless, some strengths of the SARA are clear: It is a flexible, clinically informative measure that can be used in a wide variety of contexts. It possesses acceptable predictive validity and excellent convergent validity. Its widely adopted use is therefore empirically supported, although may not be as robust as generally believed. Nevertheless, there are areas that could be improved upon both in terms of psychometric properties and research breadth and quality. SARA and other IPV risk assessment development research should aim to address the limitations identified in the present critique. Namely, by confirming reliability, testing the contribution of individual items (establishing item level validity), and compiling normative data to determine validity in diverse populations.
